# How peritoneal dialysis transforms the peritoneum and vasculature in children with chronic kidney disease—what can we learn for future treatment?

**DOI:** 10.1186/s40348-022-00141-3

**Published:** 2022-05-05

**Authors:** Maria Bartosova, Sotirios G. Zarogiannis, Claus Peter Schmitt, for the Members of the International Pediatric Peritoneal Biobank, Klaus Arbeiter, Gema Ariceta, Aysun K. Bayazit, Rainer Büscher, Salim Caliskan, Rimante Cerkauskiene, Dorota Drozdz, Sahar Fathallah-Shaykh, Günter Klaus, Rafael T. Krmar, Jun Oh, Verena Peters, Uwe Querfeld, Bruno Ranchin, Peter Sallay, Betti Schaefer, Christina Taylan, Sara Testa, Johann VandeWalle, Enrico Verrina, Karel Vondrak, Bradley A. Warady, Yok Chin Yap, Ariane Zaloszyc

**Affiliations:** 1 Center for Pediatric and Adolescent Medicine, University of Heidelberg, Heidelberg, Germany; 2 Department of Physiology, Faculty of Medicine, University of Thessaly, Larissa, Greece

**Keywords:** Chronic kidney disease, Peritoneal dialysis, Peritoneal membrane, Vascular disease, Glucose degradation products, Endothelial, Mesothelial

## Abstract

Children with chronic kidney disease (CKD) suffer from inflammation and reactive metabolite-induced stress, which massively accelerates tissue and vascular aging. Peritoneal dialysis (PD) is the preferred dialysis mode in children, but currently used PD fluids contain far supraphysiological glucose concentrations for fluid and toxin removal and glucose degradation products (GDP). While the peritoneal membrane of children with CKD G5 exhibits only minor alterations, PD fluids trigger numerous molecular cascades resulting in major peritoneal membrane inflammation, hypervascularization, and fibrosis, with distinct molecular and morphological patterns depending on the GDP content of the PD fluid used. PD further aggravates systemic vascular disease. The systemic vascular aging process is particularly pronounced when PD fluids with high GDP concentrations are used. GDP induce endothelial junction disintegration, apoptosis, fibrosis, and intima thickening. This review gives an overview on the molecular mechanisms of peritoneal and vascular transformation and strategies to improve peritoneal and vascular health in patients on PD.

Peritoneal dialysis (PD) is a life-saving kidney replacement therapy taking advantage of the properties of the peritoneum as a semipermeable biomembrane facilitating the clearance of excessive water, solutes, and toxins in patients with kidney failure. PD is the preferred and most widely used modality in children with chronic kidney disease (CKD), especially in infants and young children in whom a permanent vascular access for hemodialysis can be very challenging. Automated cycler dialysis overnight provides high compatibility with social life and schooling. Moreover, since diseases requiring chronic kidney replacement therapy are rare, only few specialized pediatric dialysis centers are available in most countries; distances are often long, and thrice weekly follow-ups as required for hemodialysis are hardly feasible in many children. PD is also preferred when there are contraindications to the use of systemic anticoagulants as required in hemodialysis and in patients with cardiovascular instability, which may be aggravated in hemodialysis by the extracorporeal blood volume and high rates of fluid removal (ultrafiltration) within few hours [1]. In adults, early survival is superior with PD as compared to hemodialysis [2].

The standardized outcomes in nephrology (SONG) initiative united patients, caregivers, and health care professionals to identify core outcomes. The SONG-PD initiative identified PD-related infections, cardiovascular disease, mortality, PD technique failure, and life participation as core outcome parameters, which should be reported in every PD-related clinical trial [3]. Efficiency and sustainability of the PD therapy, i.e., preservation of the peritoneal membrane integrity and function is relevant for all of these outcomes. Unfortunately, current PD fluids are bioincompatible, containing high amounts of glucose, and glucose degradation products (GDP) negatively impacting on local parietal peritoneal integrity and systemic health, e.g., arterioles not directly exposed to PD fluids. Cardiovascular events may already occur in early adulthood and are the primary cause of death later in life [4]. In-depth understanding of the PD-induced peritoneal and vascular pathophysiology is essential to improve patient outcome. Children are uniquely suited for sensitive and specific studies of the underlying molecular mechanisms of peritoneal and vascular transformation, since they are largely devoid of lifestyle and aging alterations, and the underlying disease in the majority of cases is limited to the kidney and urinary tract. The present review summarizes recent findings on PD-associated parietal peritoneal damage as well as the associated effects on systemic vascular disease, their underlying molecular pathomechanisms, and therapeutic strategies to mitigate these untoward effects.

## The parietal peritoneum, a semipermeable biomembrane, chronically exposed to toxic PD fluids

The parietal peritoneal membrane covers the abdominal wall and the intraperitoneal organs. It is composed of the mesothelial cell monolayer lining the submesothelial interstitial space, which reaches the underlying muscle, fat, and organ tissue. The submesothelium consists of collagen fibers and contains blood capillaries and lymphatic vessels together with nerves and sparse tissue resident inflammatory cells. The peritoneal submesothelial thickness in individuals with normal kidney function is age dependent, with mean values of 230 μm in infants to 400 μm in puberty and 170 μm in late adulthood [5]. Blood capillary density follows a U-shaped curve with an about 2-fold higher density in infants and late adulthood as compared to adolescents. Peritoneal lymphatic vessel density is low across age groups. Blood and lymphatic vessels and nerves are organized in three layers within the submesothelial space. In the first two layers, capillaries are present together with lymphatics and nerve bundles, while arterioles with about 100 μm diameter are mostly found in the third, deep submesothelial layer [5].

PD fluids contain sodium, chloride, and magnesium at low physiological concentrations and calcium at concentrations of 1.25–1.75 mmol/l. High concentrations of calcium in dialysate allow for compensation of ultrafiltration-associated calcium losses and serve the demand of the growing skeleton. The buffer compound, lactate or bicarbonate, is present at concentrations of 34–35 mmol/l, and corrects metabolic acidosis of CKD. Glucose is present at far supra-physiological concentrations of up to 4250 mg/dl to establish a crystalloid osmotic gradient to remove water. Excess electrolytes and small, water-soluble uremic toxins diffuse across the concentration gradient into the PD fluid, together with the dissolved solutes in the ultrafiltrate (convective purification). Clearance of larger, middle molecules is substantially lower; and protein-bound toxins are hardly cleared [6, 7] and their effective removal largely depends on residual kidney function. The capillary endothelium is considered the main barrier of exchange in PD, while the submesothelial interstitium is not a barrier unless major fibrosis has occurred [8]. The barrier function of the mesothelial cell monolayer is uncertain.

## The peritoneal membrane and vasculature in CKD G5

In children with CKD grade 5 prior to dialysis only minor changes of the peritoneal membrane develop [9]. These include infiltration of isolated CD45 positive inflammatory cells into the submesothelial space and peritoneal fibrin deposits. Only few mesothelial cells can be detected in the submesothelial space. These have undergone epithelial to mesenchymal transition (EMT) and acquired a migratory phenotype. Microvessel density is increased by 30%, mainly due to an increase in blood vessel density, while lymphatic vessel density is unchanged. Compared to age-matched healthy children, the abundance of transforming growth factor-ß (TGF-ß) effector molecule pSMAD2/3, reflecting pro-fibrotic signaling, is increased, while the peritoneal abundance of the pro-angiogenic cytokine vascular endothelial growth factor (VEGF) is comparable. Peritoneal vessels already exhibit mild but significant vascular lumen obliteration, even in younger children; a striking finding which is in line with vascular imaging studies in children with CKD [10]. Vessels of patients with CKD exhibit arteriosclerotic lesions, lumen obliteration, and vascular calcifications. Numerous classical risk factors such as hypertension, smoking, hypercholesterolemia, obesity, and CKD-specific factors such as accumulating uremic toxins and CKD mineral bone disease result in accelerated vascular disease. Early mechanisms include endothelial damage and dysfunction [11], which are reflected on a molecular level by reorganization of intercellular endothelial junctions, which ensure proper polarization and function, of adherens junctions and the actin cytoskeleton, and ultimately result in endothelial cell loss [12]. In medium size arteries obtained from children with CKD G5, gene sets involved in extracellular matrix organization including elastin, collagen type I, versican and tissue inhibitor of matrix metalloproteinase 2, were downregulated, and calcification-related genes osterix, runt-related transcription factor 2, and cyclin-dependent kinase Inhibitor 2A were upregulated as compared to children with normal kidney function. Tissue calcium content was substantially increased [13]. Altogether, these profound pathophysiological alterations present in children with CKD demonstrate the need for an in-depth and comprehensive understanding of the molecular pathomechanisms of vascular disease in children with CKD and, the focus of the present review, of the specific impact of PD.

## Transformation of the parietal peritoneal membrane in the course of PD

Conventional, acidic, single chamber PD fluids are still used in many countries including the UK and the US. Next to high concentrations of glucose they contain numerous highly reactive GDP generated during the heat sterilization process and prolonged storage, such as methylglyoxal and 3,4-dideoxyglucosone-3-ene (3,4-DGE). In a landmark paper, Williams et al demonstrated progressive loss of the peritoneal mesothelial cell monolayer, submesothelial fibrosis, and vascular lumen narrowing with chronic PD [14]. Compared to the few healthy controls, but not to patients with CKD G5, who exhibited some increase in vascularization [9], vessel number per mm section length was increased only in subgroups of patients requiring surgical interventions and in patients with ultrafiltration failure [14].

The underlying molecular mechanisms of the parietal peritoneal alterations induced by high GDP PD fluids were studied in rodents. Daily exposure to GDP fluids increased peritoneal and vascular peritoneal deposition of advanced glycation end products (AGE), AGE-associated RAGE activation, and resulted in peritoneal cell apoptosis, fibrosis, and angiogenesis [15]. Fibrosis-related pathways include the induction of TGF-ß and connective tissue growth factor signaling together with inflammation and EMT. Numerous pharmacological modulators targeting the pro-fibrotic and angiogenic processes have been proposed and included interference with AGE signaling by neutralizing anti-RAGE antibodies [16], cyclooxygenase-2 inhibitors, renin-angiotensin system inhibitors, and bone morphogenic protein-7 [17]. The translation into the human setting, however, is challenging due to putative systemic untoward effects [18].

Twenty years ago, neutral pH, double-chamber PD fluids were introduced, which separate glucose at very low pH from the buffer. GDP formation is largely prevented, after mixture of both compartments prior to instillation into the peritoneal cavity, pH is neutral to physiological. In vitro and experimental in vivo studies suggest less peritoneal GDP and AGE accumulation, improved local host defense, reduced mesothelial damage, less submesothelial fibrosis, and angiogenesis, i.e., better preservation of the peritoneal membrane. A recent study in mice, however, demonstrated major cell infiltration, namely pro-inflammatory M1 macrophages and interleukin-17 expressing CD4 cells, with low GDP fluids compared to high GDP fluid [19]. Thus, the improved biocompatibility profile with lower exposure to toxic GDP increased the inflammatory response to the PD fluids, containing high concentrations of glucose.

In humans, few observational studies compared the peritoneal membrane transformation with low and high GDP PD fluids. Eighty-four children on low GDP PD fluids were systematically compared with regard to histomorphometric changes and molecular mechanisms over time on PD to 90 children with CKD G5 (time of catheter insertion). Within only 4 months of PD, the peritoneal vessel density was twofold higher as compared to CKD G5. VEGF-A abundance was increased and significant infiltration of alpha smooth muscle actin positive activated fibroblasts, CD45-positive leukocytes, CD68-positive macrophages, and EMT cells developed [9]. Activation of TGF-ß-induced pSMAD2/3 and submesothelial thickening were less pronounced; significant fibrosis developed with long-term low GDP PD and was present in most patients after more than 4 years of PD. In multivariable analyses, the peritoneal vessel density independently predicted peritoneal transport function (D/D_0glucose_ and D/P_creatinine_). Submesothelial fibrosis was predicted by PD duration and the presence of EMT, peritoneal blood microvessel density by dialytic glucose exposure [9]. Different from patients treated with high GDP PD fluids, the impact of peritonitis episodes on peritoneal histomorphology [20] and transport function appear less pronounced [21], and the clinical severity of peritonitis episodes was reduced [22, 23].

The finding of early and major increase in peritoneal blood vessel density with glucose-rich, low GDP fluids available for solute and glucose transport, compared to largely unchanged vessel density in patients treated with high GDP PD fluids, is well in line with functional data obtained in the BalANZ trial [23]. During the first year of PD, patients randomized to a low GDP, neutral pH fluid had higher solute transport rates measured every 3 months and lower ultrafiltration rates than patients on high GDP, acidic PD fluids. These differences vanished thereafter. Overall PD membrane function was stable in the low GDP fluid patient group, while increasing transport status with lower ultrafiltration rates developed with long-term use of high GDP fluids. Similar functional differences over time were demonstrated in further trials comprising a total of 430 patients reflecting PD fluid-specific morphological alterations (Table 1) [21, 24]. Studies in adults systematically comparing the impact of high vs. low GDP PD fluids on peritoneal membrane demonstrated less peritoneal AGE deposition, better preservation of the mesothelial cell layer, and less peritoneal vasculopathy with low GDP PD fluids [25–27]. In children, high GDP PD fluid usage was associated with more submesothelial fibrosis, lower peritoneal blood vessel density, and less inflammatory cell invasion [27].

**Table 1:** Overview on peritoneal histomorphology in children with normal renal function and chronic kidney disease grade 5 and in children on peritoneal dialysis (PD) with PD fluids containing low and high concentrations of glucose degradation products (GDP). *EMT* epithelial-to-mesenchymal transition

Health	CKD G5	Low GDP PD	High GDP PD
Intact mesothelial monolayer	Mesothelium largely preserved	Reduced mesothelial cell coverage	More mesothelial cell loss compared to low GDP PD
Three layers containing blood, lymphatic vessels, and nerves	Vessel density mildly increased	Major and diffuse increase in submesothelial vessel density within few months of PD	Less submesothelial angiogenesis
Age-dependent vascularization and submesothelial thickness	Isolated submesothelial inflammatory cells, sparse EMT cells	Major submesothelial inflammatory cell invasion, inflammatory cytokines. Some EMT cells	Less submesothelial inflammatory cell invasion, less cytokine induction, but pronounced EMT
		Slow but steady increase in submesothelial fibrosis	More pronounced submesothelial fibrosis
	Moderate signs of vasculopathy	Dialytic glucose exposure-related vascular complement activation	Increased vascular AGE deposition, GDP exposure-related endothelial cell apoptosis, junction disruption, and endothelial cell loss. More lumen narrowing (intima thickening)

Next to the GDP content, the PD buffer compound may affect peritoneal membrane long-term function. A small size randomized trial in children demonstrated better preservation of ultrafiltration capacity per gram of dialytic glucose exposure with a pure bicarbonate versus lactate buffered low GDP fluid [28]. In vitro, the bicarbonate-buffered low GDP PD fluid induced less endothelial angiogenesis than the lactate-based fluid. The bicarbonate fluid increased the angiopoietin 1/2 ratio, i.e., induced a shift towards vessel maturation, as well as a tyrosine kinase receptor translocation to the endothelial cell membrane, where it co-localized with VE-cadherin, promoting endothelial maturation. In line with the in vitro findings, peritoneal vessels from children treated with bicarbonate PD fluids exhibited larger cross-sectional area reflecting vessel maturation, as compared to age- and glucose exposure-matched children treated with the respective lactate PD fluid [29].

Icodextrin-based solutions are an alternative to glucose-based PD solutions for a single long dwell per day. A recent Cochrane meta-analysis reconfirmed increased ultrafiltration rates, fewer episodes of fluid overload together with a reduced daily glucose absorption, which altogether probably decreases the mortality risk in adults [30]. No morphological differences were observed in peritoneal biopsies from patients using low GDP fluids together with icodextrin fluids [9, 31], but the patient numbers studied were low and the sensitivity of icodextrin specific peritoneal effects with once daily administration is probably low. In vitro, significant damage to mesothelial cells has been demonstrated with icodextrin exposure. In patient-derived mesothelial cells and in peritoneal biopsies from pediatric patients, mesothelial αB-crystallin (CRYAB) is specifically up-regulated in response to icodextrin PD fluid and correlated with markers of angiogenesis and fibrosis [32]. Altogether, there is strong evidence from randomized clinical trials that once daily administration of icodextrin for the long dwell improves ultrafiltration, hydration status, and blood pressure and by this probably decreases mortality risks. Ex vivo and in vitro studies, however, do not unanimously support local peritoneal biocompatibility of icodextrin fluids, respective long-term studies are required.

Amino acids represent another, GDP-free alternative to glucose, which in addition may improve nutritional status of PD patients, if administered at a one-to-three ratio together with glucose-based solutions. Similar to icodextrin, they are applied once daily to prevent the risk of acidosis and azotemia. In adults, several studies suggested improved protein synthesis together with maintenance of the nitrogen balance [33], but a recent meta-analysis including 14 studies, of which nine were randomized, did not demonstrate consistent improvement in anthropometric measures and yielded a slight decline in serum albumin with amino-acid fluid usage [34]. In view of the limited effect of amino acid-based PD fluids on nutritional status and the critical importance of adequate nutrition, enteral tube feeding is usually preferred in young children [35]. The impact of amino acid fluids on the peritoneal membrane has not been studied systematically. In vitro, mesothelial cell transcriptome analyses demonstrated up and downregulation of 464 genes within 24 h of incubation with amino acid-based PD fluids, as compared to 208 with high GDP PD fluid and 45 to 71 with different low GDP PD fluids. Regulated genes were mainly related to cell cycle processes [36]. In rats, amino acid containing PD fluid induced less inflammation and angiogenesis compared to glucose containing PD fluid [15, 37]. In a randomized crossover study in six patients on automated PD, effluent IL-6 was increased in day time effluent after an overnight dwell with amino acid solution [38]. The long-term effect of amino acid fluids on the peritoneal membrane is uncertain.

## PD fluid associated systemic vascular disease

GDP are rapidly absorbed from the peritoneal cavity and increase systemic AGE concentrations. Switching from high to low GDP fluids reduces circulating AGE concentrations by 20% in children [39] and adults [40], and vice-versa. There is increasing evidence that the additional reactive metabolite load associated with high GDP PD fluids has major untoward systemic effects. Several randomized trials demonstrated superior preservation of residual kidney function with low as compared to high GDP fluid usage, a key predictor of outcome [40]. Reduced glomerular, tubular, and/or vascular damage should have developed. Vascular molecular pathomechanisms have recently been demonstrated in children on PD with low and high GDP PD fluids matched for age, dialysis vintage and dialytic glucose exposure in omental arterioles protected from direct PD fluid effects by surrounding fat tissue and thus representing systemic vascular disease [27]. These arterioles underwent meticulous microdissection from the fat tissue and underwent transcriptome- and proteome-analyses. Groups were matched for age, PD vintage, dialytic glucose exposure, and history of peritonitis, while dialytic GDP exposure was 10-fold higher with GDP-rich PD fluids. GDP-rich PD fluids resulted in threefold higher arteriolar AGE concentrations, upregulation of cell death/apoptosis pathways and suppression of cell viability/survival, cytoskeleton organization, and immune response biofunctions. Vasculopathy-associated canonical pathways concordantly regulated on gene and protein level with high GDP exposure included cell death/proliferation, apoptosis, cytoskeleton organization, metabolism and detoxification, cell junction signaling, and immune response. Validation in parietal peritoneal arterioles, exposed to GDP and the high glucose concentrations of PD fluids, in independent cohorts reconfirmed tight junction disruption in single-molecule cluster analyses and endothelial cell apoptosis. Endothelial cell number per endothelial surface length is inversely correlated with the dialytic GDP exposure, with AGE, RAGE, IL-6, and p16 abundance. TGF-β–induced phosphorylation of pSMAD2/3 correlated positively with IL-6, p16, and AGE/RAGE. ZO-1 abundance correlated inversely with lumen narrowing, i.e., intima thickening [27]. Independent of PD, junctional disruption and breakdown of the endothelial barrier at atherosclerosis-prone regions has been demonstrated to enable the subendothelial accumulation of atherogenic lipids [41, 42]. In the same direction, the actin cytoskeleton plays a key role in regulating the stability of endothelial cell contacts and vascular permeability with cytoskeleton reorganization being the basis of vascular remodeling and vasculopathy. The formation of contractile stress fibers, e. g. under inflammatory conditions, contributes to junction destabilization [43] and the initiation and progression of atherosclerotic process. In vitro, we demonstrated reduction of antiapoptotic Lamin-A/C and membrane ZO-1 assembly by 3,4-DGE, while pSMAD2/3, ionic, and 4 and 10 kDa dextran permeability of arterial endothelial cells increased [27]. Altogether, these findings strongly suggest less systemic vascular disease with reduced dialytic GDP exposure.

Vascular molecular pathomechanisms in children on low GDP PD, however, are still distinct from those with CKD G5. Compared to CKD G5, we demonstrated marked omental arteriolar complement activation, which in the parietal peritoneum correlated with dialytic glucose exposure, arteriolar TGF-ß induction, and lumen narrowing [44].

## Steps forward to improve clinical outcome

**Fig. 1:**
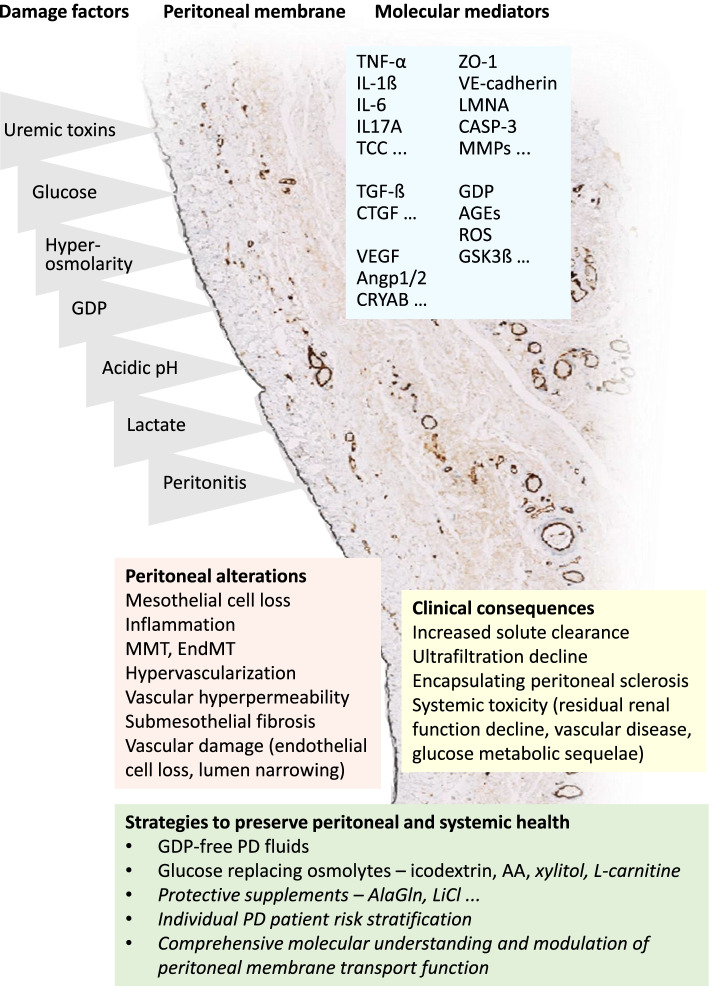
Peritoneal dialysis associated local and systemic damage and protective strategies. Reactive metabolites accumulating with chronic kidney disease combined with the unphysiological composition of current PD fluids exert major local peritoneal and systemic damage, which compromise PD long-term function and contribute to accelerated patient aging. Strategies to mitigate these PD-related sequelae are given in the lower box; in italic strategies currently under development. AA, amino acids; AGEs, advanced glycation endproducts; AlaGln, alanyl-glutamine; Angp1/2, angiopoietin 1/2; CASP-3, caspase; CRYAB, crystallin alpha B; CTGF, connective tissue growth factor; EndMT, endothelial-mesenchymal transition; GDP, glucose degradation products; GSK3ß, glycogen synthase kinase-3 beta; IL, interleukin; LiCl, lithium chloride; LMNA, lamin A; MMPs, metalloproteinases; MMT, mesothelial-mesenchymal transition; ROS, reactive oxygen species; TCC, terminal complement complex; TGF-ß, transforming growth factor ß; TNF-α, tumor necrosis factor α; VE-cadherin, vascular endothelial cadherin; VEGF, vascular endothelial growth factor; ZO-1, zonula occludens 1

There is increasing evidence that PD fluids low in GDP content have less long-term detrimental effects on the peritoneal membrane morphology [26, 45] and better maintain long-term peritoneal membrane function [23, 24, 46]. Switching from high to low GDP fluids in several countries was associated with a reduced incidence of encapsulating peritoneal sclerosis, a life-threatening complication of long-term PD [47, 48]. Next to the reduced local peritoneal damage, increasing evidence points to systemic benefits of the reduced dialytic GDP exposure. These comprise superior preservation of residual kidney function with low GDP fluid usage [49] and distinct molecular pathomechanisms overall resulting in less pronounced vascular disease. Whether these advantages translate in improved long-term PD patient outcome, however, still needs to be demonstrated. Prospective studies confirming improvement of clinically relevant end-point with low GDP fluids are still lacking. In contrast, replacement glucose by icodextrin for one long dwell per day reduces not only fluid overload, but has been shown in randomized trials to improve PD patient survival. Altogether, introduction of novel PD fluid types has been a major step forward to improved PD biocompatibility, albeit without solving the urgent need of an inert osmotic agent, devoid of local peritoneal and systemic toxicity, entirely replacing glucose. To achieve adequate ultrafiltration patients are still exposed to exceedingly high dialysate glucose concentrations of 1300–2500 mg/dl) in the majority of exchanges, and in case of reduced ultrafiltration capacity even up to 4250 mg/dl. This creates a severely diabetic milieu in the peritoneal cavity and triggers massive local and systemic damage as described above and in Table 1 and Fig. 1 [9, 44, 45]. Whether the benefits in PD treatment achieved thus far result in superior PD patient outcome as compared to hemodialysis is uncertain, since hemodialysis treatment has improved at the same time, e.g., by the introduction of high convective flow hemodialysis in children [50, 51]. Randomized comparisons of both optimized dialysis modes are lacking.

Several alternative osmotic solutions are under development. L-carnitine is a water-soluble, chemically stable compound which has a similar osmotic capacity as glucose, essentially involved in mitochondrial fatty acid oxidation and energy production. In vitro, carnitine has reduced impact on mesothelial and endothelial cells [52, 53] and in rabbits, it preserves the peritoneum better than bicarbonate glucose and icodextrin PD fluids [54]. A randomized controlled clinical trial demonstrated improved insulin sensitivity, thus making this PD solution a promising therapy especially in diabetic patients [55]. Xylitol is a five-carbon sugar alcohol, pentitol, a sugar substitute of glucose used in parenteral nutrition. In vitro studies suggest a superior mesothelial and endothelial cell viability, less mesenchymal transdifferentiation, reduced oxidative stress, reduced inflammatory cytokine secretion, reduced endothelial tube formation, and preserved mesothelial barrier function [52]. Larger clinical trials are underway combining various concentrations of xylitol with L-carnitine and low concentrations of glucose within one bag (NCT04001036 and NCT03994471). In preclinical trials taurine, a sulfonic beta-amino acid, and hyperbranched polyglycerol induced less untoward effects, in the case of the latter including better preservation of peritoneal membrane and kidney function together with less adverse systemic metabolic effects, but translation into clinical trials is pending [33].

Another strategy to mitigate the negative impact of glucose-based PD solutions is adding a protective compound. Alanyl-glutamine supplementation to low GDP PD fluid yielded promising results in a phase II clinical trial. It increased mesothelial cell mass marker CA125, improved effluent cell immunocompetence, and, of particular interest, reduced dialytic protein loss and increased small solute clearance, altogether suggesting improved semipermeability of the PD membrane [56]. In line with this, the addition of alanyl-glutamine improved endothelial cell barrier function and upregulated tight junction abundance and clustering in vitro and upregulated sealing junction claudin-5 in mice treated with alanyl-glutamine supplemented high GDP PD fluid [57, 58]. A phase III trial is underway. Another promising PD fluid additive with beneficial effects is lithium chloride, a GSK-3ß pathway modulator, which in pre-clinical studies reduced EMT and angiogenesis via downregulation of the angiogenesis mediator, small heat shock protein CRYAB [32]. Based on experimental studies, the addition of lithium is promising, but requires extended studies on systemic absorption, accumulation, and potential long-term toxicity in patients on chronic PD.

Next to improving PD fluid biocompatibility and mitigation of the untoward effects by protective supplements, current research is focused on identifying patients at particular risk of inefficient PD therapy and cardiovascular disease. A genome wide association study identified 23 single nucleotide variants at four loci associated with peritoneal solute transport rate and an estimated heritability of peritoneal transport function of 19% [59]. During the early phase of a dwell ultrafiltration mainly occurs via the water selective channel aquaporin-1; global AQP-1 knock-out mice have 50% reduced ultrafiltration [60]. A recent large-scale landmark study demonstrated AQP1 genetic variant-dependent patient outcome. The TT AQP1 promoter variant present in 10 to 16% of PD patients, and conferring decreased AQP1 promoter activity, was associated with 20-35% lower ultrafiltration rates and a 70% increased risk of technique failure or death as compared to 35 to 47% of the patients carrying a common CC AQP-1 variant [61]. Surprisingly, AQP-1 at present is the only well-characterized peritoneal membrane transporter; other transcellular transporters such as the sodium glucose co-transporter SGLT-2 [62] have been demonstrated; and the precise role, however, is uncertain. The relative contribution of the paracellular tight junctions is unknown. These should define peritoneal membrane semi-permeability and exert major transport of solutes and water and thus represent promising therapeutic targets.

## Data Availability

Not applicable.

## Abbreviations

3,4-DGE: 3,4-Dideoxyglucosone-3-ene
AGE: Advanced glycation end products
AQP-1: Aquaporin-1
CA-125: Cancer antigen-125
CD: Cluster of differentiation
CKD: Chronic kidney disease
CRYAB: αB-crystallin
EMT: Epithelial to mesenchymal transition
GDP: Glucose degradation products
GSK-3ß: Glycogen synthase kinase-3β
IL-6: Interleukin-6
PD: Peritoneal dialysis
pSMAD2/3: Phosphorylated SMAD2/3
RAGE: Receptor for advanced glycation end products
SGLT-2: Sodium glucose co-transporter-2
TGF-ß: Transforming growth factor-ß
VE-cadherin: Vascular endothelial cadherin
VEGF: Vascular endothelial growth factor
ZO-1: Zonula occludens-1
